# MiR-770 suppresses the chemo-resistance and metastasis of triple negative breast cancer via direct targeting of STMN1

**DOI:** 10.1038/s41419-017-0030-7

**Published:** 2018-01-11

**Authors:** Yaming Li, Yiran Liang, Yuting Sang, Xiaojin Song, Hanwen Zhang, Ying Liu, Liyu Jiang, Qifeng Yang

**Affiliations:** 1 Department of Breast Surgery, Qilu Hospital, Shandong University, School of Medicine, Wenhua West Road No. 107, Ji’nan, Shandong 250012 P. R. China; 2 Pathology Tissue Bank, Qilu Hospital, Shandong University, Jinan, 250012, Shandong Province People’s Republic of China

## Abstract

Chemo-resistance and metastasis of triple negative breast cancer (TNBC) contributed the most of treatment failure in the clinic. MicroRNAs (miRNAs) have been proved to be involved in many biological processes and diseases. In this study, we aimed to determine the role of miR-770 in the regulation of chemo-resistance and metastasis of TNBC. Clinically, miR-770 was highly expressed in chemo-sensitive tissues and predicted a better prognosis of TNBC. Functionally, ectopic expression of miR-770 suppressed the doxorubicin-resistance of TNBC cell lines via regulation of apoptosis and tumor microenvironment, which was mediated by exosomes. Moreover, miR-770 overexpression inhibited the migration and invasion. Rescue of STMN1 could partly reverse the effect of miR-770 in TNBC behaviors. Furthermore, we also demonstrated that overexpression of miR-770 inhibited DOX resistance and metastasis in vivo. Taken together, our results proved that miR-770 could suppress the doxorubicin-resistance and metastasis of TNBC cells, which broaden our insights into the underlying mechanisms in chemo-resistance and metastasis, and provided a new prognostic marker for TNBC cells.

## Introduction

Breast cancer is one of the most common tumors among women, and the second leading cause of cancer-related death in the world^1^. Approximately 1 to 1.3 million cases are diagnosed every year, and about 15-20% of patients belongs to the triple negative subtype (TNBC)^2^. The TNBC was defined as a subtype which lacks of estrogen receptor, progesterone receptor, and human epidermal growth factor receptor type 2 gene expression^3^. We have previously reported that patients with TNBC have a relatively poorer outcome for the rapid proliferation, early metastasis and lack of molecular targets for treatment^4^. For TNBC patients, surgery and radiotherapy are employed routinely in a similar way as other types of breast cancer, but adjuvant chemotherapy seemed to be more important for the lack of molecular targets, which became the only systematic treatment^5^. TNBC could be chemo-sensitive particularly to cytotoxic agents such as anthracyclines and taxanes, but once the chemo-resistance developed, the cells became more aggressive and metastatic^6^. The metastasis and chemo-resistance of TNBC were the most common causes leading to the treatment failure, disease recurrence and eventual death in clinic^7^.

Currently, anthracycline-based combination chemotherapy is one of the most important front-line chemotherapeutic agents, generally solely used or combined with other drugs to treat advanced or metastasis breast cancer^8^. TNBC has been reported to be more sensitive to anthracycline-based chemotherapy compared to endocrinal positive subtypes despite more than 70% of TNBC patients have residual invasive disease after chemotherapy, which partly result from the arisen of chemo-resistance, and only as few as half of the patients may experience the benefits from chemotherapy^9–12^. Moreover, studies have reported that the arisen of chemo-resistance may contribute to the metastasis of breast cancers, which further decreased the prognosis of patients^6^. Thus, it is of great significance to explore the mechanism of chemo-resistance and metastasis.

MicroRNAs (miRNAs) are a class of small non-coding regulatory RNAs that play an important role in various biological processes, including the proliferation, metastasis and chemo-resistance of triple negative breast cancer^13,14^. Recently, several studies reported miRNAs could play a role not only inside cells but also in the tumor microenvironment^15,16^. Exosomes are 30 to 100-nm vesicles containing miRNAs, lncRNAs, proteins etc, and released by most cell types, which have been reported to have great significance in the cell-to-cell communications^17,18^. Previous studies demonstrated that exosomes could influence the chemo-resistance and metastasis of breast cancer via transportation of miRNAs^19,20^. However, though a few miRNAs have been reported, the definite molecular mechanism of miRNAs function has not been well elucidated in TNBC.

In our study, we detected the miRNAs expression in chemo-sensitive and chemo-resistant tissues by miRNA microarray, and we found miR-770 was significantly decreased in chemo-resistant group. Further experiments proved miR-770 could antagonize the chemo-resistance and metastasis via targeting of STMN1, and modify the tumor microenvironment via transportation to tumor-associated macrophage.

## Results

### MiR-770 is a prognostic biomarker in triple negative breast cancer

**Fig. 1:**
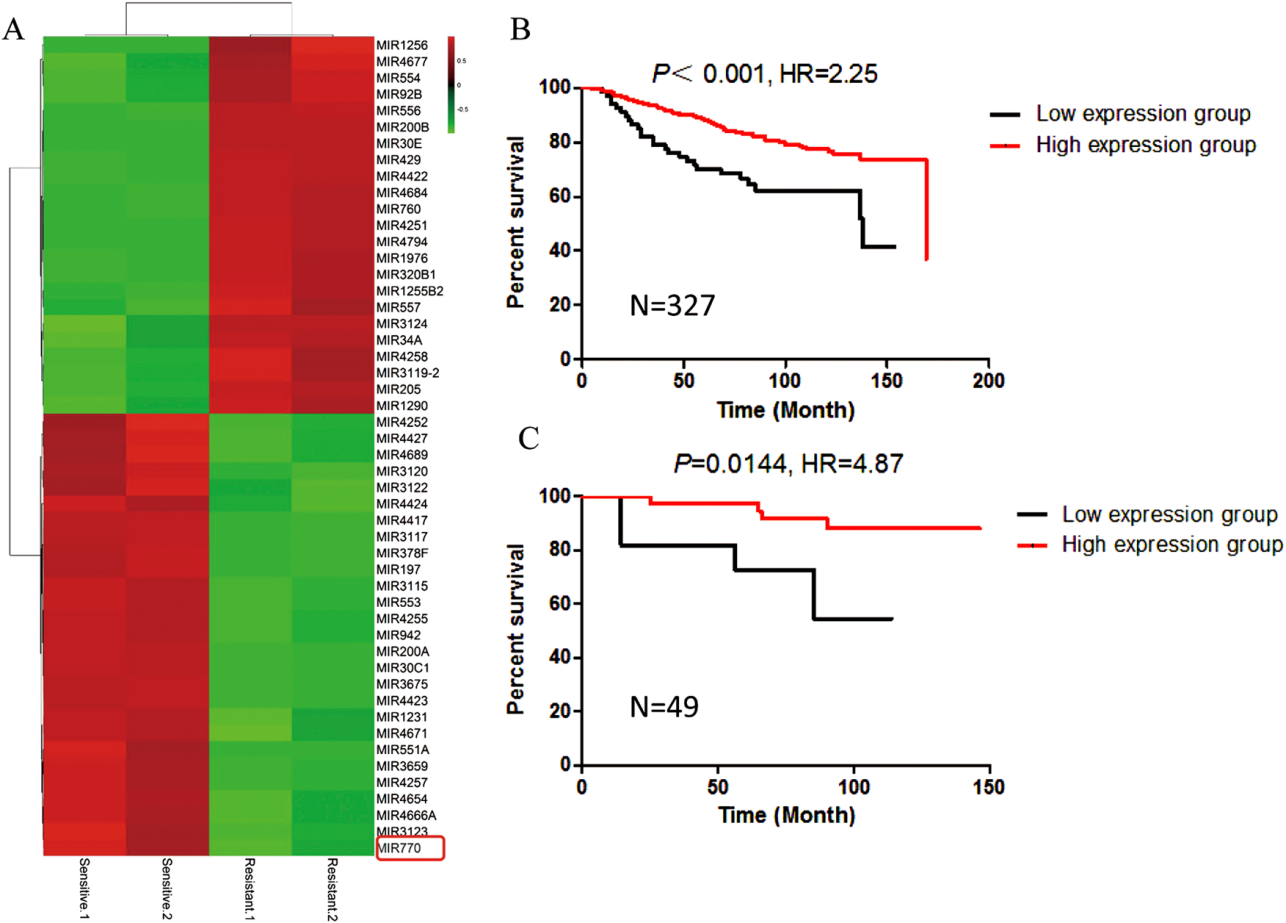
MiR-770 is aberrantly expressed in chemo-sensitive and chemo-resistant breast tissues and is prognostic. **a** Heat map diagram depicting expression of 50 miRNAs dysregulated in chemo-sensitive compared with chemo-resistant breast tissues. **b**, **c** Kaplan-Meier and Cox-regression analysis of miR-770 levels and overall survival in all **b** or TNBC **c** patients. *P < 0.05, **P < 0.01

To identify miRNAs biomarker associated chemo-resistance of TNBC, we preformed miRNA expression array in two pairs of chemo-sensitive and chemo-resistant tissues. We identified 23 miRNAs with higher expression level and 27 with lower expression level in chemo-resistant tissues with the filter of 2 fold (Fig. 1a). Among miRNAs with different expression, we found miR-770 was significantly decreased in chemo-resistance tissues, which has not been well understood in TNBC, and we focused on this miRNA in our subsequent investigations.

Next, to further determine the prognostic significance of miR-770, Kaplan-Meier survival plots and Cox regressions were computed using the datasets from GEO database (GSE20685)^21^. High expression of miR-770 contributed to a better overall survival in all type of breast cancers (*n* = 327, HR = 2.25, *P* < 0.01) and TNBC subtype (*n* = 49, HR = 4.87, *P* = 0.014) (Figs. 1b, c). These findings further demonstrated that miR-770 was a prognostic biomarker and up-regulation of miR-770 improved the prognosis of TNBC patients, which suggested that miR-770 play an important role in the regulation of chemo-resistance and progression of TNBC.

### MiR-770 promoted the chemo-sensitivity of TNBC to doxorubicin via induction of apoptosis

**Fig. 2:**
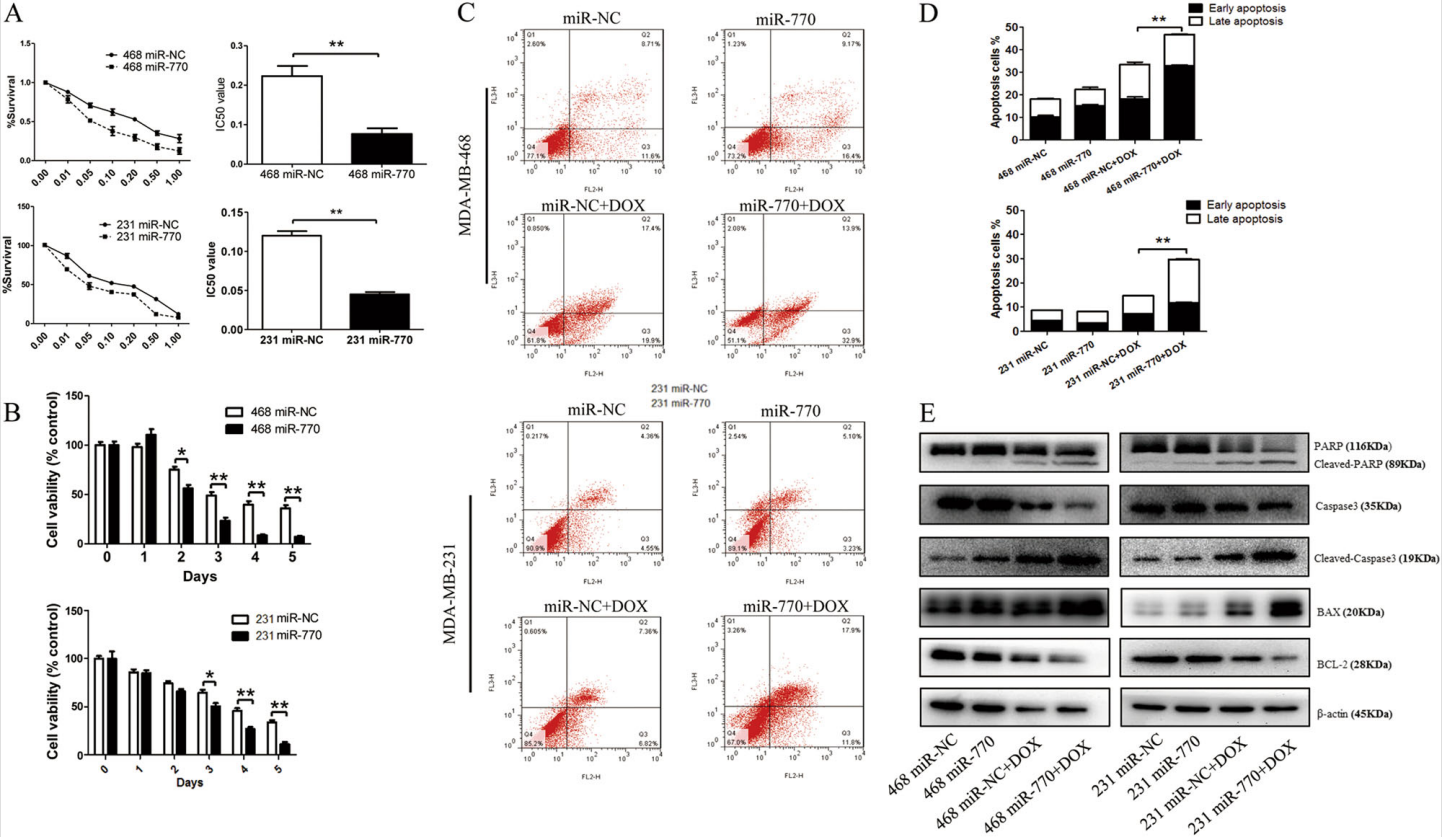
Mir-770 sensitizes TNBC cells to DOX via inducing apoptosis. **a** Effect of miR-770 overexpression in MDA-MB-468 and MDA-MB-231 cells on the IC50 value. **b** The cytotoxicity assay of special concentrations of DOX on MDA-MB-468 (0.4 μM) and MDA-MB-231 cells (0.1 μM). **c** Representative flow cytometer images of TNBC cells transfected with miR-NC or miR-770 with or without DOX treatment. The y-axis quantifies the number of cells stained with propidium iodine and the x axis quantifies number of cells stained with Annexin V-FITC. **d** Quantification of apoptosis cells of Fig. 2.C. **e** Western blot analysis of apoptosis related markers in cells transfected with miR-NC and miR-770 treated with or without DOX. Data represent means ± S.D. of at least three independent experiments. *P < 0.05, **P < 0.01

For the miRNAs expression array showed that miR-770 was decreased in chemo-resistant tissues, to further understand the mechanism of miR-770 in the regulation of chemo-resistance on DOX, we transfected MDA-MB-468 and MDA-MB-231 with miR-NC or miR-770. We firstly examined the effect of miR-770 on the IC50 of DOX in two TNBC cell lines. As shown in Fig. 2a, we found that overexpression of miR-770 could significantly decrease the IC50 of DOX in both cell lines, which suggested that miR-770 could promote the chemo-sensitivity of TNBC. Moreover, we also examined the cytotoxicity of specific concentrations of DOX on MDA-MB-468 (0.4 μM) and MDA-MB-231 (0.1 μM), and the cell viability was detected daily for 5 days. Our results proved that miR-770 could also promote the chemo-sensitivity in a time-dependent manner (Fig. 2b). What’s more, to eliminate the possibility of miR-770 may effect cell growth and cell cycle, we performed cell proliferation and cell cycle assay, and results showed that miR-770 overexpression alone do not affect cell proliferation and cell cycle (Supplementary Fig. S2).

Previous studies demonstrated that DOX could kill the TNBC cells via induction of apoptosis^22,23^. To further explore the mechanism of miR-770 on the regulation of DOX sensitivity, we detected the synergistic effect of miR-770 and DOX on the percentage of apoptosis. Results proved that the addition of miR-770 alone showed not significant effect on the apoptosis, while the combination use of miR-770 and DOX could significantly promote the DOX induced apoptosis (Figs. 2c, d). Moreover, we found that the combination use of miR-770 and DOX could also active the apoptosis pathways (Fig. 2e). These findings suggested that miR-770 could sensitize TNBC cells to DOX via activation of DOX induced apoptosis.

### MiR-770 could be transferred by exosomes and regulate TAMs induced chemo-resistance

Based on above results, we proved that miR-770 could sensitize TNBC cells to DOX via its endogenous effect on apoptosis pathway. Macrophages are one of the most important components of the stroma which shift their functional phenotypes in response to various microenvironmental signals generated from tumor and stromal cells, and was defined as tumor associated macrophages (TAMs)^24^. Previous studies have reported that the aggregation of TAMs decreased the cytotoxicity of DOX to breast cancer cells^25,26^, to further explore the influence of miR-770 overexpression on DOX sensitivity, we examined whether miR-770 could be transferred by exosomes to modify the tumor microenvironment.

**Fig. 3:**
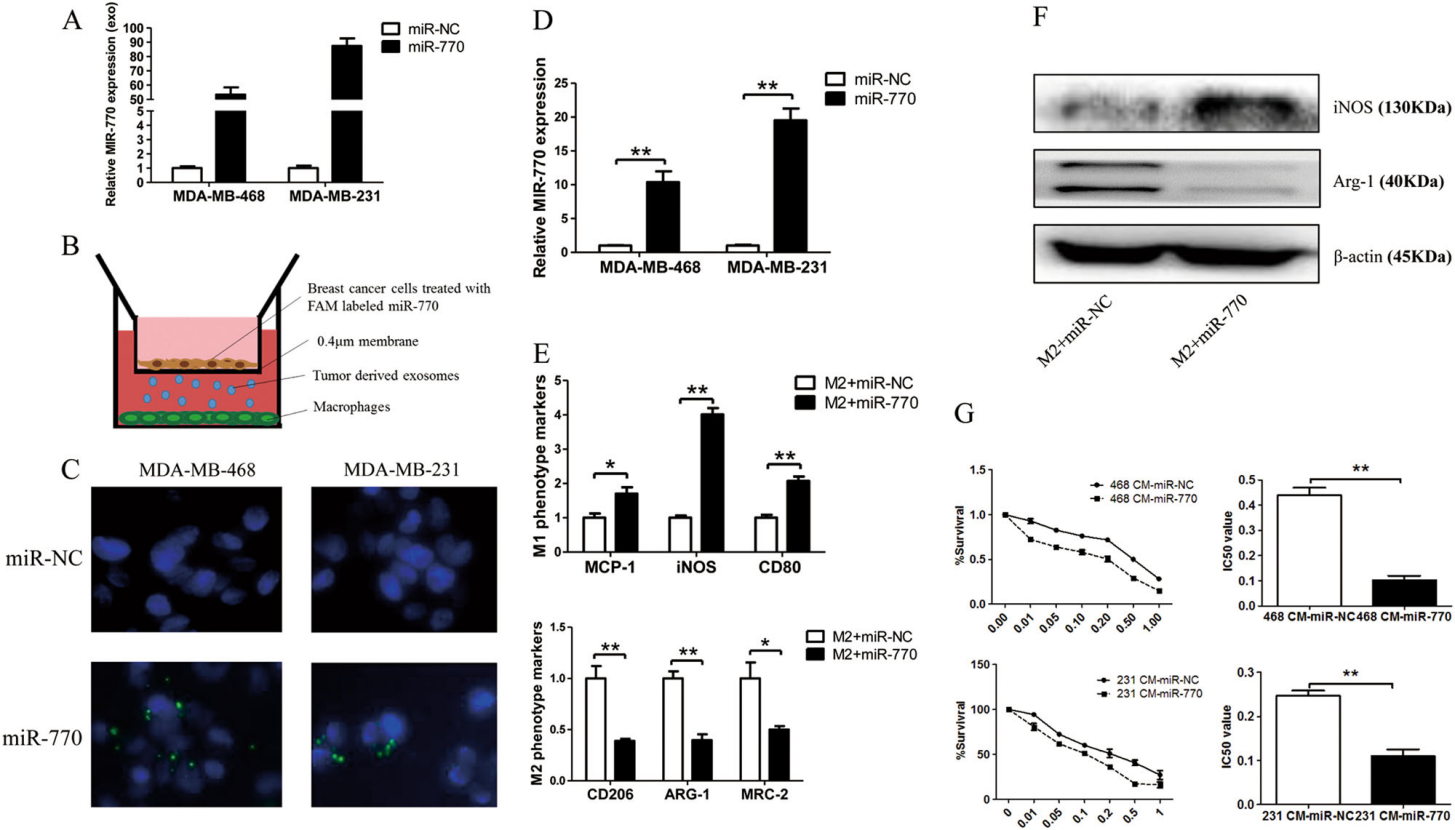
Mir-770 could be packed in TNBC derived exosomes and transferred to influence to phenotype of TAMs. **a** The expression of miR-770 in exosomes that derived from TNBC cells transfected with miR-NC and miR-770. **b** The ideograph of co-culture system. **c** MDA-MB-468 and MDA-MB-231 was transiently transfected with FAM labeled miR770 or without transfection (Control) were co-cultured with PMA treated THP-1. Fluorescence microscopy was used to detect the green signals in THP-1 cells. **d** The relative miR-770 expression in THP-1 cells co-cultured with TNBC cells transfected with miR-NC or miR-770. **e** THP-1 cells were treated with 150 nM PMA for 24 h and were transfected with miR-NC/770, then 20 ng/ml IL-4 and 20 ng/ml were added for another 24 h. The mRNA of both cells were isolated and the relative mRNA expression of M1 and M2 markers was detected. **f** THP-1 cells were treated as above, the protein was isolated and iNOS and Arg-1 were detected. **g** THP-1 cells were treated as above, then the conditioned medium (CM) was collected. The IC50 of both MDA-MB-468 and MDA-MB-231 cells to DOX was detected when cultured with 468 CM-miR-NC/770. Data represent means ± S.D. of at least three independent experiments. *P < 0.05, **P < 0.01

Now that we have proved that miR-770 could be transferred to TAMs, we explored the effect of miR-770 overexpression on TAMs phenotypes and TAMs induced chemo-resistance. After PMA treated for 24 h, THP-1 cells was transfected with miR-NC and miR-770, then IL-4 and IL-13 was added for 24 h for induction of M2 phenotype. For mRNA detection, M1 markers (MCP-1, iNOS, CD80) was significantly increased while M2 markers (CD206, ARG-1, MRC-2) was remarkable decreased in miR-770 group (Fig. 3e). Then the detection of protein levels proved the same result (Fig. 3f). These results suggested that miR-770 may participate in the process of macrophage polarization, which promoted M1 phenotype and inhibited M2 phenotype. We then determined the effect of miR-770 overexpression in THP-1 cells on TAMs induced chemo-resistance. As shown in Fig. 3g, the conditioned medium from THP-1 transfected with miR-770 (CM-miR-770) could antagonize the chemo-resistance induced by M2 phenotype THP-1 transfected with miR-NC (CM-miR-NC), the IC50 was significantly decreased in both TNBC cells treated with CM-miR-770. In conclusion, our results demonstrated that the overexpression of miR-770 could be transferred from TNBC cells to TAMs and regulate the polarization of macrophages, thus sensitizing TNBC cells to DOX.

### MiR-770 inhibited the migration and invasion ability via modification of EMT pathway

**Fig. 4:**
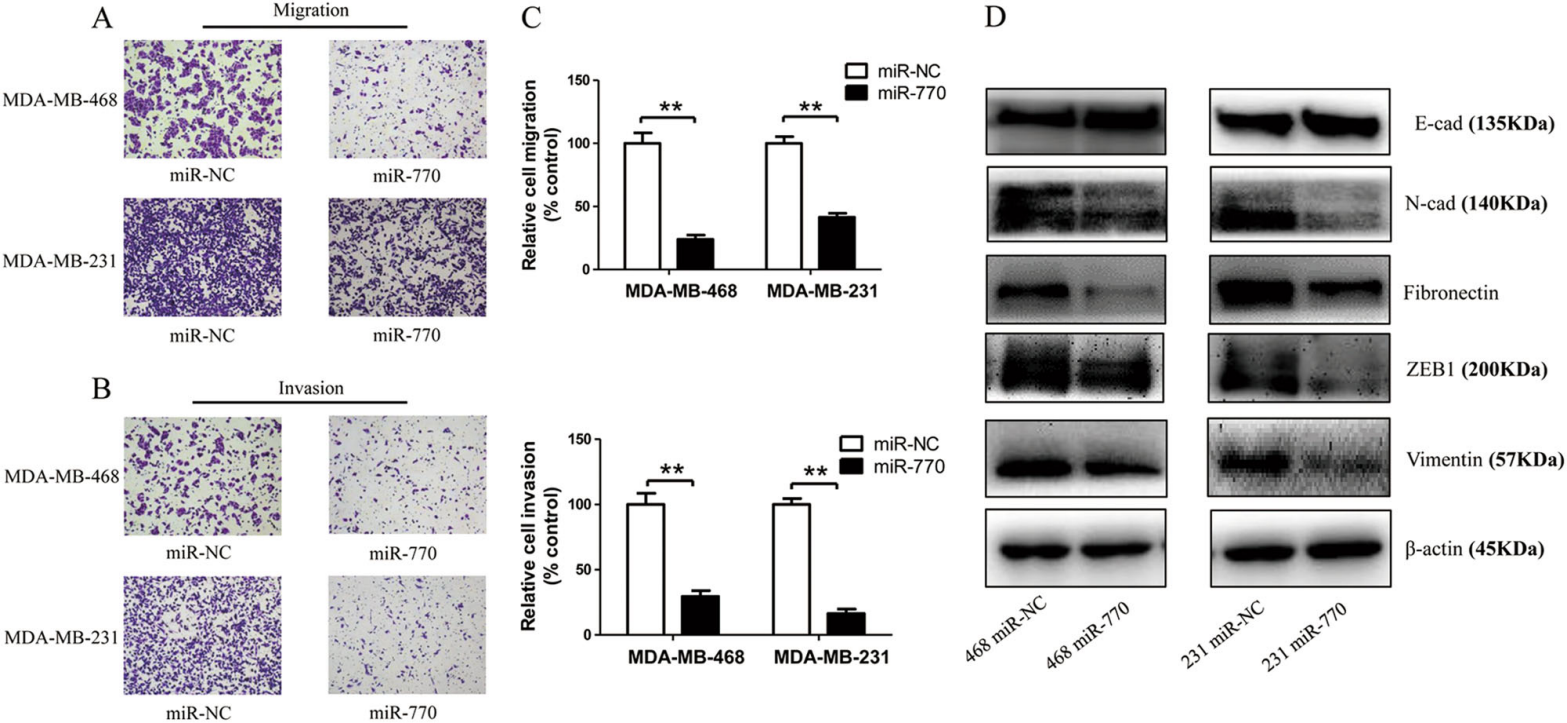
MiR-770 inhibits aggressive behaviors of TNBC cells. **a**, **b** Effect of miR-770 overexpression on the metastatic **a** and invasive **b** ability of MDA-MB-468 and MDA-MB-231 cells. Cells were counted after staining with crystal violet. Representative images are shown. **c** Quantification of migration and invasion of cells transfected with miR-770 compared with control. **d** Western blot analysis of EMT related markers in cells transfected with miR-NC and miR-770. Data represent means ± S.D. of at least three independent experiments. *P < 0.05, **P < 0.01

Previous studies reported that the arisen of chemo-resistance may associated with the high metastatic ability of breast cancer cells^27^. To investigate the role of miR-770 on the metastatic ability of TNBC cells, we transfected MDA-MB-468 and MDA-MB-231 cells with miR-NC or miR-770, and transwell system was used to examine the change of migration and invasion ability. As shown in Figs. 4a–c, the cell counts of MDA-MB-468 and MDA-MB-231 were significantly decreased in both migration and invasion systems after transfection with miR-770 mimics, which suggested miR-770 could inhibit the metastatic ability of TNBC cells. Further exploration of the mechanism of miR-770, we found that miR-770 could modify the EMT pathway, which promoted epithelial phenotypes and inhibited mesenchymal phenotypes (Fig. 4d). In conclusion, miR-770 could inhibit the migration and invasion of TNBC cells via inhibition of EMT pathway.

### Knockout of miR-770 promoted the chemoresistance and metastasis of TNBC cells

To further demonstrated the important role of miR-770 in the regulation of chemoresistance and metastasis, we knockout miR-770 with miR-770 inhibitor. In accordance with results above, we proved that knockdown of miR-770 could promote DOX resistance in both MDA-MB-468 and MDA-MB-231 cell lines, and increase the ability of migration and invasion. Results were shown as Supplementary Fig. S1 in supplementary materials.

### STMN1 is a direct target of miR-770

**Fig. 5:**
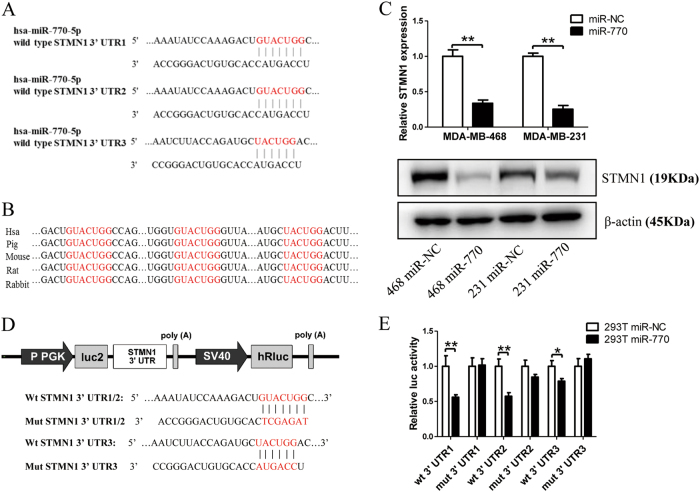
STMN1 mRNA is a direct target of miR-770. **a** The three predicted targeting sequence of miR-770 of the STMN1 3′ UTR. **b** Sequence alignment of the predicted miR-770 seed region in the 3′ UTR of STMN1 from 5 organisms. **c** qRT-PCR and western blot assay revealed that overexpression of miR-770 level significantly reduced STMN1 expression in MDA-MB-468 and MDA-MB-231 cells. **d** The diagram illustrates the construction of the luciferase reporter plasmids. The substitutions of nucleotides within the three binding sites were shown and the three constructs named as mut1, mut2, and mut 3 separately (Wt: wild type; Mut: mutant type.). **e** Luciferase reporter assay in HEK293T cells revealed miR-770 suppressed STMN1 3′ UTR luciferase activity of all wide-type constructs. Firefly luciferase activity was normalized to the internal Renilla luciferase activity. Data represent means ± S.D. of at least three independent experiments. *P < 0.05, **P < 0.01

To explore the underlying molecular mechanism of miR-770-mediated chemo-sensitivity and metastasis suppression, we performed in silico studies to search for potential gene targets of miR-770 using the bioinformatics algorithms: TargetScan, miRDB, and PICTAR5. All three algorithms predicted STMN1 as a target of miR-770^28^; the putative targets sequence are in base pairs 54-60, 267-273, 409-415 of the STMN1 3′ UTR (Fig. 5a). Homology searches revealed that this putative binding site is evolutionarily conserved (Fig. 5b).

To further analysis the role of miR-770 on the expression of STMN1, the mRNA and protein level of STMN1 was examined after the transfection of miR-770. Our results showed that the overexpression of miR-770 could significantly decrease the STMN1 expression in both mRNA and protein level, our results suggested that miR-770 might regulate the STMN1 expression via targeting of 3’ UTR (Fig. 5c).

To determine whether miR-770 could bind directly to the 3′ UTR of target mRNA, we cloned both three wild type and mutant binding sites of miR-770 into the pmiRGLO vector, respectively, which was synthesized from Vigene Biosciences (Fig. 5d). For luciferase activity assays, miR-NC or miR-770 was co-transfected with pmiRGLO-3′ UTR vectors into HEK293T cells. As shown in Fig. 5e, the relative luciferase activity was remarkably decreased by miR-770 when the three wild-type 3′ UTR of STMN1 was present, which 3′ UTR1 and 3′ UTR2 exerted a stronger repression effect compared with 3′ UTR3. In addition, when transfected with mutant vectors which contained the STMN1 3′ UTR with mutations in the putative miR-770 binding sites, miR-770 mimic caused no obvious change in luciferase activity. Taken together, these findings indicated that STMN1 mRNA is a direct, down-stream target of miR-770 in breast cancer cells.

### The tumor suppressor role of miR-770 is mediated by down-regulation of STMN1

**Fig. 6:**
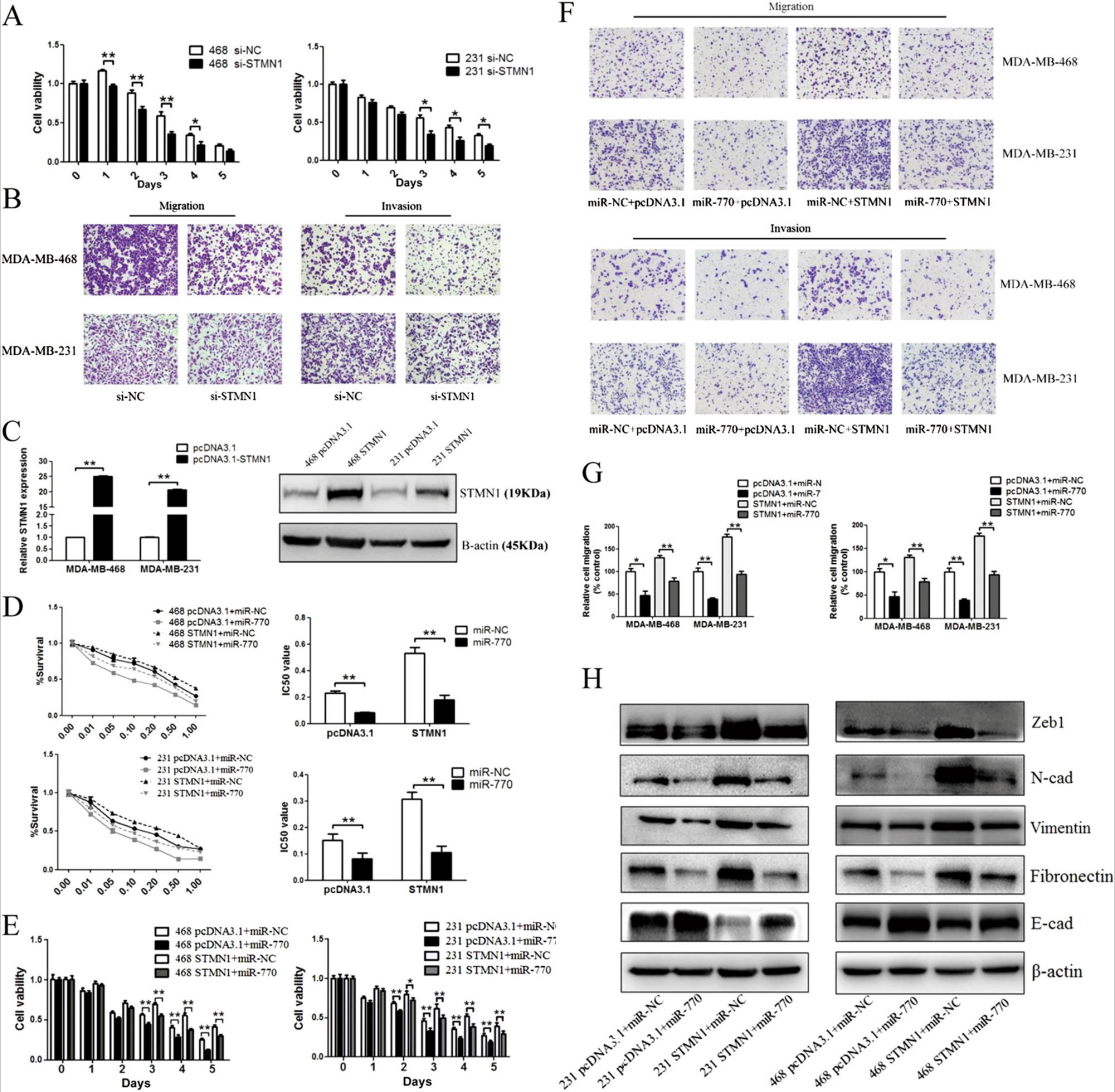
Tumor-suppressive effect of miR-770 is mediated by targeting of STMN1 mRNA. Both MDA-MB-468 and MDA-MB-231 cells were transfected with miR-NC or miR-770 together with either STMN1 or pcDNA3.1. **a** mRNA and protein level were detected for verification. And IC50 assay **b** and cytotoxicity test **c** were performed. The migration **d** and invasion ability of TNBC cells. **e** Quantification of migration and invasion of cells. Data represent means ± S.D. of at least three independent experiments. *P < 0.05, **P < 0.01

STMN1 has been proved as the direct target of miR-770, to evaluate whether STMN1 was a functional target gene, we firstly interfered its expression via specific siRNA. As shown in Figs. 6a, b, knockdown of STMN1 significantly decreased the ability of both DOX resistance and metastasis. Based on this finding, we further determine whether miR-770-induced inhibition of chemo-resistance, migration and invasion could be reversed by restoration of STMN1 expression, and we performed a gain-of-function assay. Specifically, we transfected a vector expressing STMN1 without its 3′ UTR, which resulted in constitutive expression of STMN1 without the potential for miR-770-mediated degradation, and the overexpression of STMN1 was verified by qRT-PCR and western blot (Fig. 6c). Then the pcDNA3.1 or pcDNA3.1-STMN1 was co-transfected with miR-NC or miR-770. As shown in Fig. 6d, we also proved that STMN1 could antagonize miR-770 induced decrease of IC50 in both TNBC cell lines. Moreover, STMN1 could significantly reverse the miR-770 induced repression of chemo-resistance in a time dependent manner. (Fig. 6e).

Then we explored the role of STMN1 on the inhibition of metastatic ability caused by miR-770. Results showed that the overexpression of STMN1 could reverse the miR-770 induced inhibition of both migration and invasion ability in MDA-MB-468 and MDA-MB-231 cell lines (Figs. 6f, g). Moreover, we further proved overexpression of STMN1 could reverse the miR-770 induced inhibition of EMT pathway (Fig. 6h). These results reinforced that STMN1 mRNA is a direct functional target of miR-770.

### MiR-770 suppressed DOX resistance and metastasis in a xenograft model

**Fig. 7:**
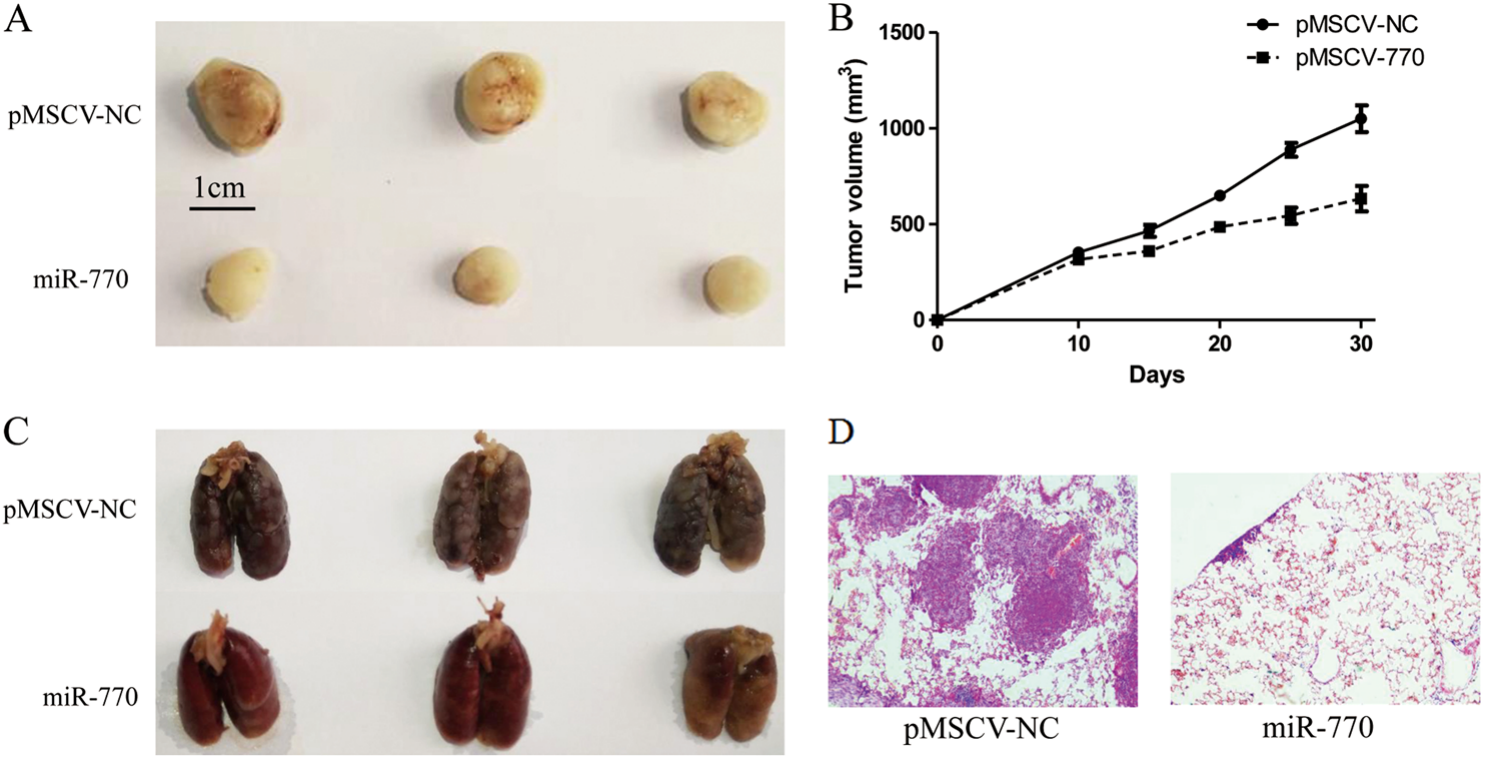
MiR-770 overexpression inhibited DOX resistance and metastasis in xenograft models. **a**, **b** MiR-770 synergized with DOX to suppress the growth of tumor. **c** Overexpression of miR-770 inhibited lung metastasis of MDA-MB-231 cells. **d** Representative images of hematoxylin and eosin (HE)-staining of lungs isolated from mice that received tail vein injection of MDA-MB-231 pMSCV-NC cells and pMSCV-770 cells

**Fig. 8:**
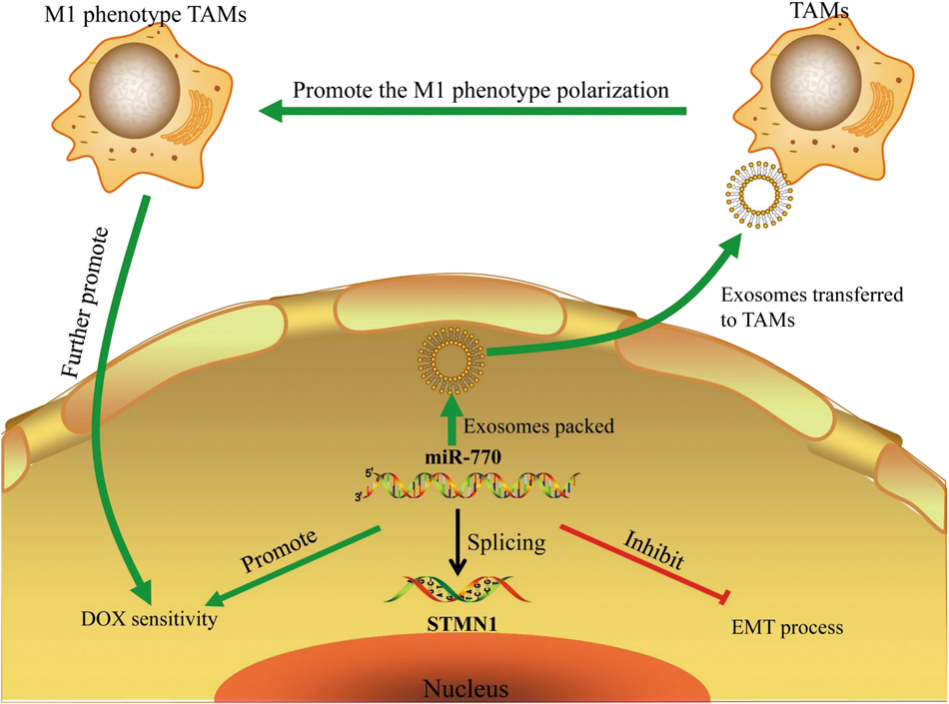
MiR-770 could significantly inhibit the DOX resistance and metastasis of triple negative breast cancer cells, which is mediated via regulation of apoptosis and EMT and modification of tumor associated macrophages

Our results have proved that overexpression of miR-770 inhibited DOX resistance and metastasis of TNBC cell lines in vitro. To further validated the role of miR-770, MDA-MB-231 was chosen to be transfected with pMSCV-NC and pMSCV-770 for its highly tumorigenic and metastatic abilities. The chemoresistance and metastasis of both pMSCV-NC and pMSCV-770 cell lines were validated. As shown in Figs. 7a, b, both pMSCV-NC and pMSCV-770 cell lines were seed subcutaneously, and mice were injected with saline or DOX (1 mg•kg-1) at day 5, 10, 15, 20 and 25. Our results demonstrated that tumors in pMSCV-770 group were significantly shrunken compared with pMSCV-NC group. Previous studies have proved that lung metastasis was an indicator of tumor aggressiveness. As shown in Fig. 7c, the metastasis of pMSCV-770 overexpression group was significantly decreased than the pMSCV-NC group, which was also demonstrated by HE staining assay (Fig. 7d). Our results have approved that over-expression of miR-770 could inhibit chemo-resistance as well as metastasis of TNBC in vivo Fig 8.

## Discussion

The TNBC was firstly reported in 2006, which was defined as a subtype with a more aggressive behavior and poorer prognosis^4^. The research on TNBC never stopped for the aggressive clinical behavior and lack of molecular target. The chemo-resistance and metastasis were two of the most important causes leading to the relapse and eventually death of TNBC patients, and numerous studies have focused on this aspect^29^. MiRNAs have been reported to take part in the biological process of tumor development including chemo-resistance and metastasis, and a lot of miRNAs have been found to play an important role in this process. Niu et al. found that miRNA-181a could function as a tumor promoter via direct suppression of BAX, and the overexpression of miRNA-181a contributed to the chemo-resistance and metastasis of TNBC cells^30^. Hong et al. reported that miR-125 could inhibit the EMT process and chemo-resistance and TNBC cells via targeting of MAP2K7^31^. These studies suggested that miRNAs might play an important role in regulating of chemo-resistance and metastasis of TNBC.

Mir-770 has been found to be correlated with kinds of cancers, including gastric cardia adenocarcinoma, lung cancer and ovarian cancer^32–34^. Zhao et al. reported that miR-770 was down-regulated in cisplatin resistant OVC patients and could predict the response to cisplatin. Overexpression of miR-770 promoted chemo-sensitivity of cancer cells via inhibition of DNA repair^34^. Moreover, Lee et al. proved that miR-770 sensitized cancer cells to radiation via targeting of PDZ-binding kinase^33^. However, the role of miR-770 in TNBC still remained unknown. In this study, we explored the significance of miR-770 on the chemo-resistance and metastasis of TNBC and whether miR-770 could function via exosomes to modify the tumor microenvironment.

Based on miRNA microarrays, we demonstrated that some miRNAs were different expressed in chemo-resistant TNBC and chemo-sensitive patients, and miR-770 was one of the most significantly changed miRNAs which high expression predicted more sensitive to chemotherapy. What’ more, the data from TCGA suggested that miR-770 could also serve as a prognostic marker which down-regulation of miR-770 predicted a poorer prognosis. To understand the mechanism of miR-770 in the regulation of chemo-resistance of DOX, we performed cytotoxicity test and we found that overexpression of miR-770 could decrease the IC50 of both MDA-MB-468 and MDA-MB-231 cell lines, and promote the sensitivity to DOX in a dose dependent manner. Further studies we validated that miR-770 could promote DOX induced activation of apoptosis, while miR-770 alone showed no significant effect on the apoptosis pathway, which proved that miR-770 could sensitize TNBC cells to DOX through promoting apoptosis. The metastasis and chemo-resistance have been reported to be closely related, the arisen of chemo-resistance could promote metastasis of cancer cells and the metastasis contributed to chemo-resistance^35–37^. After transfection of miR-770 mimics, we found that the metastasis and invasion ability were significantly decreased in both TNBC cell lines, and western blotting showed the process of epithelial-mesenchymal transition was inhibited. One critically important, yet often overlooked, component of tumor progression is the tumor microenvironment, which is primarily composed of fibroblasts, extracellular matrix proteins, endothelial cells, macrophages and so on^38^. The tumor microenvironment has been reported to be correlated with the proliferation, metastasis, and chemo-resistance of cancer cells. One of the most important components tumor microenvironment was macrophages which could shift their phenotypes to influence the behavior of tumor cells^39^. And we evaluated the effect of miR-770 on TAMs. We found that miR-770 could be packed into exosomes, and transfection of miR-770 in MDA-MB-468 and MDA-B-231 cells increased its expression in tumor derived exosomes. Co-culture assay proved that miR-770 in tumor derived exosomes could be transferred into TAMs, and increased the expression of miR-770 in macrophages. Further experiments found that high expression of miR-770 influenced the polarization of macrophages which promote M1 phenotype and inhibit M2 phenotype. Finally we evaluated whether up-regulation of miR-770 in TAMs influencing TAMs induced chemo-resistance, and we found the IC50 was significantly decreased in both TNBC cell lines cultured with miR-770-CM and compared with miR-NC-CM. In conclusion, we proved that miR-770 suppresses the chemo-resistance and metastasis of triple negative breast cancer via regulation of apoptosis and EMT and modification of tumor microenvironment.

Stathmin1 (STMN1), also known as oncoprotein 18, is an important cytoplasmic phosphoprotein that regulates cellular microtubule dynamics, which has been reported to take part in many types of cancers^40^. STMN1 promotes microtubule depolymerization by sequestering tubulin and stimulating catastrophes^41,42^. The overexpression of STMN1 has been reported to be negatively associated with the prognosis of many cancers including nasopharyngeal carcinoma^43^, hepatocellular carcinoma^44^, colorectal cancer^45^ and so on. What’ more, studies proved that up-regulation of STMN1 could promote the proliferation, metastasis, and chemo-resistance of cancers, which the expression of STMN1 alone could be a prognostic indicator^46–48^. In our study, we proved that miR-770, a potential tumor suppressor, could specifically target and down-regulate STMN1, thus inhibit the metastasis and chemo-resistance of TNBC cells. Moreover, studies about STMN1 also focused on its role in macrophage polarization, Xu et al. reported that down-regulation of STMN1 is required for the phenotypic changes and classical activation of macrophages. This study also confirmed our discovery that miR-770 overexpression is associated with M1 polarization of macrophages. In a word, we proved that the tumor suppression effect of miR-770 was mediated by targeting of STMN1, which has been demonstrated as a tumor promoter in kinds of cancers.

In conclusion, we demonstrated that miR-770 could significantly inhibit the DOX resistance and metastasis of triple negative breast cancer cells, which is mediated via regulation of apoptosis and EMT and modification of tumor microenvironment (Fig. 7). Conversely, overexpression of STMN1, which is a direct functional target of miR-770, promoted breast cancer cell DOX resistance and metastasis. The newly identified miR-770/STMN1 axis provides novel insight into the chemo-resistance and metastasis of triple negative breast cancer, and represents a potential therapeutic target for the treatment of triple negative breast cancer.

## Materials and methods

### Ethics statement and human tissue samples

All experimental procedures were approved by the Ethical Committee of Shandong University. Breast tissues were obtained at the time of surgery from patients admitted to Qilu hospital, and immediately stored at −80 °C until use. All patients provided written informed consent for the use of these clinical materials in research.

### Cell lines and cell culture

All cell lines were obtained from the American Type Culture Collection (Manassas, VA), and were maintained using standard media and condition. Briefly, MDA-MB-231 and MDA-MB-468 were culture with Dulbecco’s modified Eagle’s medium (Invitrogen, Carlsbad, CA, USA) containing 10% fetal bovine serum (Hyclone). THP-1 were were maintained in Roswell Park Memorial Institute (RPMI) 1640 medium supplemented with 10% fetal bovine serum and 5 mg/ml insulin. All cell lines were grown at 37 °C in a 5% CO2 cell culture incubator.

### Cell transfection with miRNA and plasmids

MiR-770 mimic, miR mimic control, miR-770 inhibitor and inhibitor control were purchased from GenePharma (Shanghai, China). For miR-770 overexpression, plasmids was constructed from Vigene Biosciences (Rockville, USA). For STMN1 interference, siRNA and its control were synthesized from GenePharma (Shanghai, China). For STMN1 overexpression, vector was constructed as described previously^49^. Briefly, STMN1 cDNA was cloned into the multiple cloning sites of vector pcDNA3.1 (Invitrogen, Carlsbad, CA, USA), and the resultant expression vector and empty vector were transfected into MDA-MB-468 and MDA-MB-231 cells to establish STMN1 overexpressing and control cell lines, respectively. Sequence of siRNAs and primers for plasmid construction are shown in the Supplementary Table S1. Cells were seeded in 60 mm dishes (6 × 10^5^ cells/dish) and transfected using Lipofectamine 2000 (Invitrogen) for further assays.

### Cell viability assay

Cell viability was determined by a 3-(4, 5-dimethylthiazol-2-yl)-2, 5-diphenyltetrazolium bromide (MTT) assay. MDA-MB-468 and MDA-MB-231 cells were cultured in 96-well plates, after incubation overnight, the medium was replaced with solutions containing different concentrations of DOX (0, 0.01, 0.05, 0.1, 0.2, 0.5, 1 μM). After incubation for the indicated time, 20 μl of MTT (5 mg/ml in PBS) was added into each well and incubated for 4 h. The supernatants were carefully aspirated, and 100 μl of dimethyl sulfoxide was added to each well. Absorbance values at 490 nm were measured on a Microplate Reader (Bio-Rad).

### Migration and invasion assays

Migration and invasion assays were performed as described previously using the Transwell system (Corning Costar, Lowell, MA, USA)^50^. In the migration assay, 700 μL of medium with 20% Fetal bovine serum (FBS) was added to the lower well of each chamber and 1 × 10^5^ cells suspended in serum-free medium were added to the upper inserts. After incubation for the indicated time, the total number of cells adhering to the lower surface of the membrane was quantified in six representative fields. The invasion assay was performed in the same way as the migration assay except that the membrane was coated with matrigel (BD Biosciences, Bedford, MA, USA).

### Cell apoptosis assay

Cell apoptosis was detected using an Annexin V-FITC/PI apoptosis detection kit (JingMei Biotech, Beijing, China) according to the manufacturer’s protocols. Briefly, cells were treated with EDTA-free trypsin), and resuspended with 400 μL Annexin binding buffer at a concentration of 10^6^ cells/ml. Then, 5 ul FITC-conjugated AnnexinV and 10 ul PI were added to the cells and incubated at room temperature for 15 min in dark. Cell apoptosis assay was performed within 1 h post-staining on a flow cytometry.

### Isolation of exosomes

After cell cultures reached 90% confluency, cells were washed with PBS and incubated with freshly prepared complete medium containing exosome-free FBS for 48 h. then exosomes were isolated with a polyethylene glycol-based method as previous described with little modification^51^. Vesicle-containing medium from cell culture was centrifuged at 500×*g* for 5 min and followed by 2000×*g* for 30 min to remove cellular debris and large apoptotic bodies. Then the media was added to an equal volume of a 2 × PEG solution and samples were mixed thoroughly by inversion, and incubated at 4 °C overnight (at least 12 h). The next day, samples were centrifuged in a tabletop centrifuge at 10,000×*g* for 1 hour at 4 °C to acquire the pellet of exosomes.

### In vitro detection of miR-770 transfer

For the co-culture experiments, MDA-MB-468 and MDA-MB-231 cells were transfected with FAM-labeled miR-770 (GenePharma, Shanghai, China) or without transfection (Control) and were grown on the 0.4 μm pore size transwell (Thermo Fisher Scientific, Carlsbad, CA, USA), then THP-1 cells treated with 150 nM PMA and grown on the coverslips in the bottom well of the transwell. After 24 h of transfer, THP-1 cells were fixed with 4% paraformaldehyde and the nuclei were stained with 4,6-diamidino-2-phenylindole blue. Fluorescent microscopy was used to detect the green signals in the THP-1 cells.

### Quantitative RT–PCR analysis

Total RNA was extracted from cells using the TRIzol reagent (Invitrogen, Carlsbad, CA, USA). PrimeScript reverse transcriptase (RT) reagent kit (TaKaRa, Shiga, Japan) was used to synthesize cDNA from total RNA. MiRNA from 1 μg of total RNA was reverse transcribed using the Prime-Script miRNA cDNA Synthesis Kit (TaKaRa, Shiga, Japan). Real-time PCR was performed on an Applied Biosystems StepOne plus Real-Time PCR System. Primer information used in the study is provided in the Supplementary Table S1.

### Western blot analysis

Western blot analysis was performed as described previously [17]. In brief, cells were harvested and lysed in lysis buffer (1 × PBS, 1% NP40, 0.1% sodium dodecyl sulfate, 5 mM EDTA, 0.5% sodium deoxycholate and 1 mM sodium orthovanadate) containing protease inhibitors. Subsequently, 50 μg of total cellular protein from each sample were separated by 10% SDS-PAGE and electro-transferred onto polyvinylidene fluoride (PVDF) membrane using a semi-dry blotting apparatus (Bio-Rad, Hercules, CA, USA). The membranes were blocked with 5% nonfat milk at room temperature for 1 h, and then incubated with primary antibodies overnight at 4 °C. After incubation with the appropriate secondary antibodies, the protein bands were detected using the Pro-lighting HRP agent. Expression of β-actin was used as a loading control. The antibody used in this study was obtained from Cell Signaling Technology (Beverly, MA): E-Cadherin (24E10), N-Cadherin (D4R1H), ZEB1 (D80D3) Vimentin (D21H3), PARP (46D11), Caspase-3 (9662), Bax (D2E11), Bcl-2 (D17C4), STMN1 (3352); Santa Cruz Biotechnology (CA): ARG-1 (sc-4496), INOS (sc-649), Fibronectin (sc-29011).

### Dua-luciferase reporter assay

The dual-luciferase miRNA Target Expression vector pmirGLO (Promega, Madison, WI, USA) was used to generate luciferase reporter constructs. Three 3′ UTR of STMN1, containing different putative miR-770-binding sites were amplified and cloned into pmirGLO. Mutant reporter plasmids were constructed as described previously^52^. Cells were seeded in 96-well plates and co-transfected with wild-type or mutated STMN1 3′ UTR constructs, and miR-770 mimic or negative control. Luciferase activity was measured with the dual-luciferase reporter assay system (Promega). Firefly luciferase activity was normalized against Renilla luciferase activity.

### In vivo chemo-resistance and metastasis assay

The in vivo chemo-resistance and metastasis assay was performed as described previously^50^. Briefly, MDA-MB-231 vector and MDA-MB-231 miR-770 cells (1 × 10^7^cells) in 200 μl of PBS: Matrigel (1:3, v/v)) were injected subcutaneously into left flank of 4-6 weeks-old BALB/c nu/nu female mice (five mice per group), and mice were injected with saline or DOX (1 mg•kg-1) at day 5, 10, 15, 20, and 25. Tumor growth rate was monitored by measuring tumor diameters every 5 days. Both maximum (L) and minimum (W) length of the tumor were measured using a slide caliper, and the tumor volume was calculated as ½LW^2^. When mice were killed, tumors were collected. To produce experimental lung metastasis, 5 × 10^5^ cells were injected into the lateral tail veins of 4–6 weeks-old BALB/c nu/nu female mice (seven mice per group). After 2 weeks, all the mice were killed under anesthesia. The lungs were collected and fixed in 10% formalin. For tissue morphology evaluation, hematoxylin and eosin (HE) staining was performed on sections from embedded samples as previously described^53^. All animal experiments were performed with the approval of Shandong University Animal Care and Use Committee.

### Statistical analysis

All data are presented as means ± S.D. from at least three independent experiments. The software SPSS V18.0 was used for statistical analysis. Statistical significance of differences between two groups was evaluated using Student’s *t*-test, and one-way ANOVA was used to determine the significance of differences among multiple groups. Differences with *P* < 0.05 were considered statistically significant.

## Electronic supplementary material

Supplementary Figures

Supplementary Table
