# Supplementary material for: MiR-770 suppresses the chemo-resistance and metastasis of triple negative breast cancer via direct targeting of STMN1
Source: Cell Death Dis. 2018 Jan 11;9(1):14. doi: 10.1038/s41419-017-0030-7 (PMC5849036; doi:10.1038/s41419-017-0030-7)
Supplement: Supplementary file 1 — Supplementary Figures [file 41419_2017_30_MOESM1_ESM.docx]

### Supplementary Figure S.1


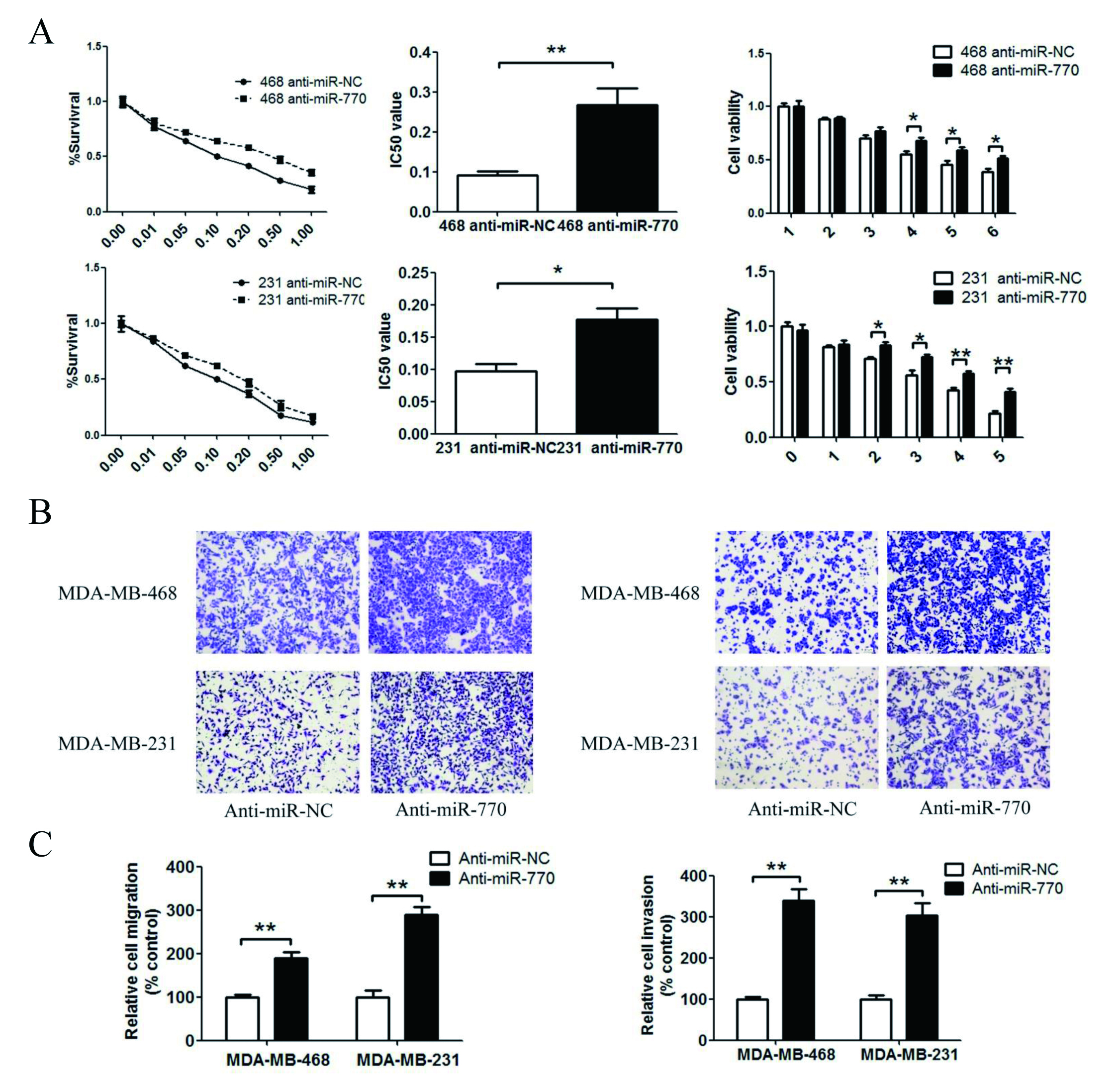


**Figure S1**

**Knockout of miR-770 promoted chemoresistance and metastasis of TNBC cells**. (A) Effect of miR-770 knockout in MDA-MB-468 and MDA-MB-231 cells on the promotion of IC50 value. And the cytotoxicity assay of special concentrations of DOX on MDA-MB-468 (0.4μM) and MDA-MB-231 cells (0.1μM) transfected with anti-miR-NC or anti-miR-770. (B, C) Effect of miR-770 overexpression on the metastatic and invasive abilities of MDA-MB-468 and MDA-MB-231 cells. Data represent means ± S.D. of at least three independent experiments. *P < 0.05, **P < 0.01.

### Supplementary Figure S.2


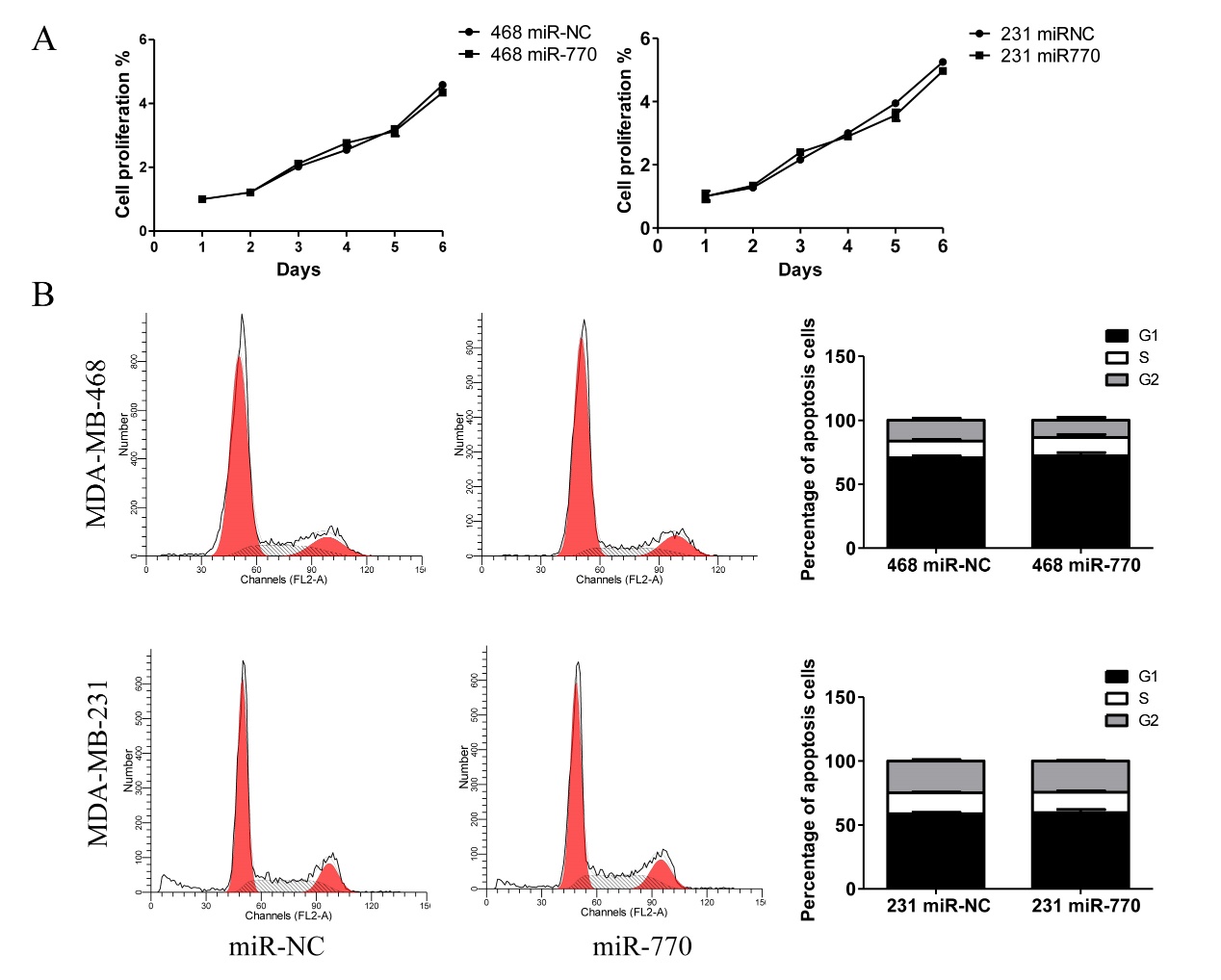


**Figure S2**

**Overexpression of miR-770 showed no effect on cell proliferation and cell cycle of TNBC cells**. (A) Proliferation of MDA-MB-468 and MDA-MB-231 cells transfected with miR-NC or miR-770 were examined with MTT assay. (B) Effect of miR-770 overexpression on cell cycle of both TNBC cell lines. Data represent means ± S.D. of at least three independent experiments.
