# Supplementary material for: MiR-770 suppresses the chemo-resistance and metastasis of triple negative breast cancer via direct targeting of STMN1
Source: Cell Death Dis. 2018 Jan 11;9(1):14. doi: 10.1038/s41419-017-0030-7 (PMC5849036; doi:10.1038/s41419-017-0030-7)
Supplement: Supplementary file 2 — Supplementary Table [file 41419_2017_30_MOESM2_ESM.docx]

**Supplementary Table S1**: Primers for plasmid construction, and qPCR

| Name | | | Sequence |
| --- | --- | --- | --- |
| Plasmid construction |  | | |
|  | pcDNA3.1-STMN1 | F | CGCGGATCCATGGCTTCTTCTGATATCCAGGT |
|  |  | R | CCGGAATTCGTCAGCTTCAGTCTCGTCAG |
|  | pMSCV-770 | F | CGCGGATCCAGTCTGAGCTGATGGCAGCAA |
|  |  | R | CCGGAATTCGACCCTCCCGGTCTTCTTC |
| qPCR | miR-770 | F | CCAGTACCACGTGTCAG |
|  |  | R | GAACATGTCTGCGTATCTC |
|  | STMN1 | F | AGAACCGAGAGGCACAAATGGC |
|  |  | R | TCTCGTCAGCAGGGTCTTTGGA |
|  | MCP-1 | F | AGAATCACCAGCAGCAAGTGTCC |
|  |  | R | TCCTGAACCCACTTCTGCTTGG |
|  | iNOS | F | GCTCTACACCTCCAATGTGACC |
|  |  | R | CTGCCGAGATTTGAGCCTCATG |
|  | CD80 | F | CTCTTGGTGCTGGCTGGTCTTT |
|  |  | R | GCCAGTAGATGCGAGTTTGTGC |
|  | CD206 | F | AGCCAACACCAGCTCCTCAAGA |
|  |  | R | CAAAACGCTCGCGCATTGTCCA |
|  | ARG-1 | F | TCATCTGGGTGGATGCTCACAC |
|  |  | R | GAGAATCCTGGCACATCGGGAA |
|  | MRC-2 | F | GGCAAGGACAAGAAGTGCGTGT |
|  |  | R | CTTTGGTGACGTTGCTGCGCTT |
